# Use of Ultrasmall Superparamagnetic Iron Oxide Enhanced Susceptibility Weighted Imaging and Mean Vessel Density Imaging to Monitor Antiangiogenic Effects of Sorafenib on Experimental Hepatocellular Carcinoma

**DOI:** 10.1155/2017/9265098

**Published:** 2017-06-21

**Authors:** Shuohui Yang, Jiang Lin, Fang Lu, Zhihong Han, Caixia Fu, Hongchen Gu

**Affiliations:** ^1^Department of Radiology, Zhongshan Hospital, Shanghai Medical College, Fudan University, Shanghai Institute of Medical Imaging, Shanghai 200032, China; ^2^Department of Radiology, Shuguang Hospital, Shanghai University of Traditional Chinese Medicine, Shanghai 200021, China; ^3^Institute of Functional and Molecular Medical Imaging, Fudan University, Shanghai 200040, China; ^4^Department of Pathology, Shuguang Hospital, Shanghai University of Traditional Chinese Medicine, Shanghai 200021, China; ^5^Siemens Shenzhen Magnetic Resonance Ltd., Shenzhen 518057, China; ^6^School of Biomedical Engineering, Med-X Research Institute, Shanghai Jiao Tong University, Shanghai 200030, China

## Abstract

We investigated effectiveness of ultrasmall superparamagnetic iron oxide enhanced susceptibility weighted imaging (USPIO-enhanced SWI) and mean vessel density imaging (*Q*) in monitoring antiangiogenic effects of Sorafenib on orthotopic hepatocellular carcinoma (HCC). Thirty-five HCC xenografts were established. USPIO-enhanced SWI and* Q* were performed on a 1.5 T MR scanner at baseline, 7, 14, and 21 days after Sorafenib treatment. Intratumoral susceptibility signal intensity (ITSS) and* Q* were serially measured and compared between the treated (*n* = 15) and control groups (*n* = 15). Both ITSS and* Q* were significantly lower in the treated group at each time point (*P* < 0.05). Measurements in the treated group showed that ITSS persisted at 7 days (*P* = 0.669) and increased at 14 and 21 days (*P* < 0.05), while* Q *significantly declined at 7 days (*P* = 0.028) and gradually increased at 14 and 21 days. In the treated group, significant correlation was found between* Q* and histologic microvessel density (MVD) (*r *= 0.753,* P <* 0.001), and ITSS correlated well with MVD (*r *= 0.742,* P *= 0.002) after excluding the data from baseline. This study demonstrated that USPIO-enhanced SWI and* Q* could provide novel biomarkers for evaluating antiangiogenic effects of Sorafenib on HCC.

## 1. Introduction

Hepatocellular carcinoma (HCC) is a highly vascular tumor. The growth and metastasis of HCC require tumor angiogenesis, which has provided a strong rationale for using antiangiogenic therapy [1]. Tumor microvessel density (MVD) is a useful index to evaluate tumor angiogenesis and its response to antiangiogenic therapy [2, 3]. However, its measurement is limited clinically because of its invasiveness and sampling bias. Therefore, noninvasive imaging techniques for tumor angiogenesis evaluation, which can be used repeatedly and accurately to monitor the status of the entire tumor vascularity, are of the utmost importance in the follow-up of these targeted treatments. With inherent complexity and variable reproducibility, several noninvasive imaging modalities, such as dynamic contrast-enhanced (DCE) CT, MRI, ultrasound, and most recently intravoxel incoherent motion, have been used to assess the functional properties of tumor angiogenesis before and after antiangiogenic therapy [4–7]. But these techniques do not demonstrate tumor macro- and microvessels themselves, nor do they allow quantification of tumor microvasculature which can be used as an imaging analogue to histological MVD. Although noninvasive CT angiography (CTA) and MR angiography (MRA) are excellent angiographic methods of visualizing larger blood vessels, they are not adequate for depiction of the smaller vasculature in the tumors due to insufficient spatial and/or temporal resolution [8–10]. Recently steady-state ultrasmall superparamagnetic iron oxide enhanced susceptibility weighted imaging (USPIO-enhanced SWI) has been successfully employed for detection of intratumoral macrovasculature in orthotopic HCC xenografts [11]. Moreover, earlier reports have shown that an MRI index *Q*, which is defined as *Q* = ΔR_2_/(Δ*R*_2_^*∗*^)^2/3^, where *R*_2_ and R_2_^*∗*^ are the spin echo and gradient-echo relaxation rate shifts caused by the injection of USPIO, was sensitive to tissue MVD [12–14]. In these studies, the measurement of* Q* was proposed as an MRI estimate of histologically derived MVD. In consideration of all these findings, we hypothesized that a combined USPIO-enhanced SWI and* Q* could be used to evaluate tumor vessels quantitatively at both macro- and microvasculature levels and to provide unique pieces of information about tumor angiogenesis.

Sorafenib, currently the only approved therapy for advanced HCC, is an oral multikinase inhibitor with profound antiangiogenic effects [5, 15, 16]. Although various imaging biomarkers have been developed with controversial results to assess the therapeutic efficacy of Sorafenib, few of them could directly reflect the alteration in the quantity of tumor neovasculature after treatment [4, 6, 7, 17, 18].

Hence, the purpose of this study was to test the feasibility and effectiveness of combined USPIO-enhanced SWI and *Q* in one-stop steady-state vessel imaging for monitoring antiangiogenic effects of Sorafenib on orthotopic HCC xenografts.

## 2. Materials and Methods

### 2.1. Animal Model and Experimental Protocol

This experimental protocol was performed with the approval of our institutional committee for animal research. Human HCC cell lines, HCC-LM3 (Liver Cancer Institute of Fudan University, Shanghai, China), were first established and cultured according to a previous report [19]. Nude mice (Shanghai Institute of Materia Medica, Chinese Academy of Sciences, Shanghai, China) with a weight of 23–25 g each were used for tumor xenograft model establishment [20]. HCC-LM3 cells (5 × 10^6^/0.2 ml/site) were inoculated subcutaneously in the left mediolateral region of axilla in the mice. When the tumor reached a diameter beyond 1 cm, it was removed and cut into tumor blocks with a volume of 1 mm^3^. A total of 35 nude mice were implanted with these tumor blocks into the left liver lobes. At the 21st day after tumor implantation, 5 out of 35 xenografts were randomly chosen for a baseline MRI. The other 30 mice were randomly assigned to either the Sorafenib treated group (*n* = 15) or the control group (*n* = 15). Mice in both groups were subdivided into 3 subgroups according to different periods at days 7 (*n* = 5), 14 (*n* = 5), and 21 (*n* = 5), respectively, after initiation of treatment. The treated group received 30 mg/kg body weight Sorafenib daily by oral gavage [21]. The control group received 0.2 ml 0.9% saline alone at the same schedule and route of administration. For the treatment solution, 200 mg Sorafenib (Bayer Schering Healthcare Pharmaceuticals, Leverkusen, Germany) was dissolved in 10 ml 99.7% ethyl alcohol and 10 ml castor oil, and followed by 60 ml 0.9% saline. After MRI studies at baseline or at each time point following treatment, mice in the corresponding groups were immediately sacrificed for histological examination.

### 2.2. MR Imaging

MR studies were performed on a clinical 1.5 Tesla MR scanner (MAGNETOM Aera; Siemens Healthcare, Erlangen, Germany) with the mice placed in prone position in an animal cradle. A 16-channel wrist coil was used for signal reception. Prior to MR imaging, anesthesia was induced by intraperitoneal injection of 3% sodium pentobarbital at a dose of 40 mg/kg body weight.

MR imaging was started with transverse *T*_1_*-*weighted Turbo spin echo (TSE) (TR = 480 ms; TE = 13 ms; field of view, 80 mm; section thickness, 2 mm; flip angle, 90°), coronal *T*_2_*-*weighted TSE (TR = 4000 ms; TE = 74 ms; field of view, 100 mm; section thickness, 2 mm; flip angle, 150°), and transverse diffusion weighted imaging (DWI) (TR = 5000 ms; TE = 64 ms; field of view, 295 mm; section thickness, 2 mm; 4* b* values: 0, 50, 400 and 800 s/mm^2^).

And then *T*_2_*-*weighted images were obtained for *T*_2_ measurement using a 2-dimensional transverse TSE sequence. Parameters were TR = 4000 ms; TE = 74 ms; field of view, 80 mm; section thickness, 2 mm; voxel size, 0.3 × 0.3 × 2.0 mm^3^; flip angle, 150°; bandwidth, 150 Hz/Px; and scan time, 1 min 58 s. For T_2_^*∗*^ measurement, transverse T_2_^*∗*^-weighted images were acquired by using a multiecho (5 echoes) gradient-echo (GRE) sequence with TEs of 3.91, 11.3, 18.5, 25.6, and 32.7 ms and a TR of 422 ms. Flip angle was 60°; bandwidth was 260 Hz/Px; and scan time was 1 min 01 s. The other scanning parameters were the same as *T*_2_*-*weighted sequence. Subsequently, all animals were bolus injected with USPIO with a dosage of 8 mg Fe/kg body weight and at a flow rate of 0.1 ml/s into tail veins [11]. With a delay of 5 minutes to allow for steady-state distribution of USPIO in the blood, *T*_2_ and T_2_^*∗*^ measurements with the same scanning parameters were performed again [11, 12].

Lastly, three-dimensional USPIO-enhanced SWI was done in transverse planes with the following parameters: TR = 30 ms; TE = 20 ms; field of view, 120 mm; section thickness, 0.9 mm; voxel size, 0.5 × 0.5 × 0.9 mm^3^; flip angle, 15°; bandwidth, 150 Hz/Px; and scan time, 1 min 38 s.

The USPIO particles were synthesized according to previous reports [22, 23]. Briefly, the crystal size of iron core is 6.1 nm and hydrodynamic size is approximately 20 nm. The* R*1 and* R*2 values in water are 8.98 and 27.90 mmol^−1^ l s^−1^ at 1.5 T. The mean plasma half-life in rats is approximately 3.77 hours.

### 2.3. SWI Evaluation

SW images including magnitude, phase, and minimum intensity projection and the final SW images were automatically reconstructed inline by MR scanner immediately after acquisition [24]. All evaluation was done on the final SW images with a commercially available workstation (Syngo Multimodality Workplace; Siemens Healthcare, Erlangen, Germany) [11, 25]. These images were evaluated randomly and independently by two experienced radiologists with 11 and 24 years' experience of MR image interpretation, respectively. They were blinded to records regarding the baseline, treatment versus control, treatment schedule, and histological examination results.

The degree of intratumoral susceptibility signal (ITSS) in the tumor on the USPIO-enhanced SW images was assessed [11]. ITSS was defined as hypointense linear or tubular form structures, or dots, or dots mixed with linear or tubular structures on contiguous slices within the tumor. The degree of ITSS was graded from 0 to 5: grade 0, no ITSS; grade 1, one to ten ITSSs; grade 2, eleven to twenty ITSSs; grade 3, twenty-one to thirty ITSSs; grade 4, thirty-one to forty ITSSs; and grade 5, forty-one or more ITSSs on the center slice through the tumor [11, 26]. Diffuse or flaky hypointensities were excluded from evaluation because the quantification of these findings could be unreliable.

### 2.4. *Q* Calculation

T_2_^*∗*^ maps were automatically generated inline by using MapIt software (Siemens Healthcare, Erlangen, Siemens). ΔR_2_^*∗*^ was computed according to ΔR_2_^*∗*^ = R_2,post_^*∗*^ − R_2,pre_^*∗*^ = 1/T_2,post_^*∗*^ − 1/T_2,pre_^*∗*^, where T_2,pre_^*∗*^ and  T_2,post_^*∗*^ are the pre- and postcontrast relaxation times [27]. Meanwhile, Δ*R*_2_ was calculated from signal intensities pre- (S_pre_) and postcontrast (S_post_) of the TSE images: Δ*R*_2_ = (1/*T*_*E*_) ln⁡ (s_pre_/s_post_) [27].

Lastly, the mean vessel density or* Q* is given by* Q* = ΔR_2_/(ΔR_2_^*∗*^)^2/3^ [12–14]. If negative Δ*R*_2_ or* Δ*R_2_^*∗*^ values were obtained, the mouse would be excluded [28].

**Figure 1:**
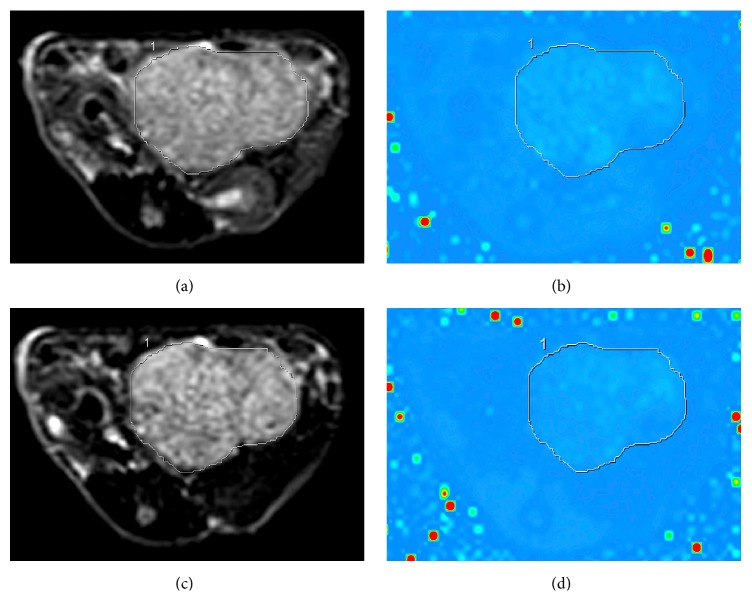
Unenhanced and ultrasmall superparamagnetic iron oxide (USPIO) enhanced *T*_2_*-*weighted and T_2_^*∗*^ mapping with superimposed region of interest in xenograft hepatocellular carcinoma. (a) Unenhanced *T*_2_*-*weighted image (tumor signal intensity = 354.2). (b) Unenhanced T_2_^*∗*^ mapping (tumor T_2_^*∗*^* = *48 ms). (c) USPIO-enhanced *T*_2_*-*weighted image (tumor signal intensity = 305.9). (d) USPIO-enhanced T_2_^⁎^ mapping (tumor T_2_^*∗*^* = *24.7 ms).

To obtain averaged information, regions of interest (ROIs) were first drawn on USPIO-enhanced *T*_2_*-*weighted images to outline the border of the tumor on the central slice and then these ROIs were copied to the corresponding unenhanced *T*_2_*-*weighted images and unenhanced and USPIO-enhanced T_2_^*∗*^ mapping images by the same radiologists who performed SWI evaluation (Figure 1) [29]. All calculations were done on the same workstation.

All the measurements of *T*_2_, T_2_^*∗*^, and ITSS were done twice at the same slice and the measured values were averaged by each radiologist. After a 4-week interval, these measurements were repeated and the final averaged values were calculated.

Tumor volume was measured on USPIO-enhanced SWI images by commercially available 3D software (Vitrea, Minnetonka, MN, USA). Free-drawing mode was used to outline the margin of the tumor on every slice and then tumor volume was automatically calculated. In order to avoid contamination by hemorrhage in the tumor, images from conventional *T*_1_*-*weighted, *T*_2_*-*weighted, and DW imaging were reviewed and compared side-by-side. If there was hemorrhage on the central slice, the most adjacent slice would be chosen for SWI and *Q* evaluations.

### 2.5. Histologic Examination

After completion of MR scanning, the nude mice in the corresponding subgroups were sacrificed by means of cervical dislocation. The tumor was harvested and fixed in a 10% buffered formalin solution for at least 24 hours and subsequently sliced in the central transverse section with a slice thickness of 2 mm to match MR images.

To determine MVD of the tumor, the slices were stained immunohistochemically for the specific endothelial antigen CD31 (Abcam, Cambridge, UK). Then all slides were fully digitalized using an Aperio ScanScope and Leica SCN400 (Leica Biosystems, Buffalo Grove, IL, USA), saved as compressed Aperio scn files, typically yielding 200–700 MB per slide. The whole slides were transferred to a PC with 1278 × 928 pixels screen resolution and evaluated by SlidePath Gateway Client software (Leica Biosystems) [30]. Four areas with the densest CD 31 positive vessels in tumor were selected as hot spots on each whole slide at lower power (4x and 10x magnification). Then vessels were manually counted twice in three higher-power fields (20x magnification) in each hot spot and results were averaged and expressed as the highest number of microvessels. The MVD measurement was done twice and results were averaged by a pathologist with 8 years of experience in histologic analysis of HCC.

To detect the distribution of iron particles in the tumor, either within tumor vessels or taken up by macrophages, both anti-CD31 and anti-CD68 (Abcam) immunohistochemistry with iron particles counterstaining were performed on one tumor model randomly selected from each treated subgroup.

### 2.6. Statistical Analysis

Tumor volume, ITSS,* Q*, and histologic tumor MVD at each time point in treated and control subgroups were compared by Mann–Whitney* U* test. Kruskal Wallis test and Mann–Whitney* U* test were used to compare ITSS and* Q* among baseline and three subgroups of both treated and control mice. Relations between ITSS,* Q,* and histologic MVD and tumor volume of all the tumors in both groups were evaluated with Spearman rank correlation test. The relationship between ITSS and *Q* was also assessed. The degree of correlation was determined by calculating correlation coefficient rho (*r*). 0 ≤ |*r*| < 0.2 was considered as poor or no correlation; 0.2 ≤ |*r*| ≤ 0.4 was fair; 0.4 < |*r*| ≤ 0.6 was moderate; 0.6 < |*r*| ≤ 0.8 was good; and |*r*| > 0.8 was defined as excellent correlation. The inter- and intraobserver agreements in quantification of ITSS,* Q,* and MVD were assessed by using kappa values and intraclass correlation coefficients (ICCs) in the treated group and baseline. Analyses were performed with Statistical Package for the Social Sciences version 18.0 (IBM, Armonk, NY, USA). Values of* P* < 0.05 were considered statistically significant. Measurement and ranked data were presented as (means ± SD) and median (P25, P75), respectively.

## 3. Results

The SWI and* Q* evaluations were successfully completed in 35 and 34 mice, respectively. One mouse from the 21-day treated subgroup was excluded for* Q* calculation because of its negative Δ*R*_2_ values. The tumors could be visualized clearly on USPIO-enhanced SWI, *T*_2_*-*weighted, and T_2_^*∗*^ mapping images. Gross intratumoral hemorrhage was seen in 1 mouse in the control group at day 21 and in 5 mice of the treated group (2 at day 7, 1 at day 14, and 2 at day 21). However, most of the hemorrhage foci were found at the edge slices of the tumor and none of them was observed on the slices used for SWI and* Q* evaluation.

### 3.1. Comparison of ITSS Scoring and* Q* between the Control and Treated Groups

**Figure 2:**
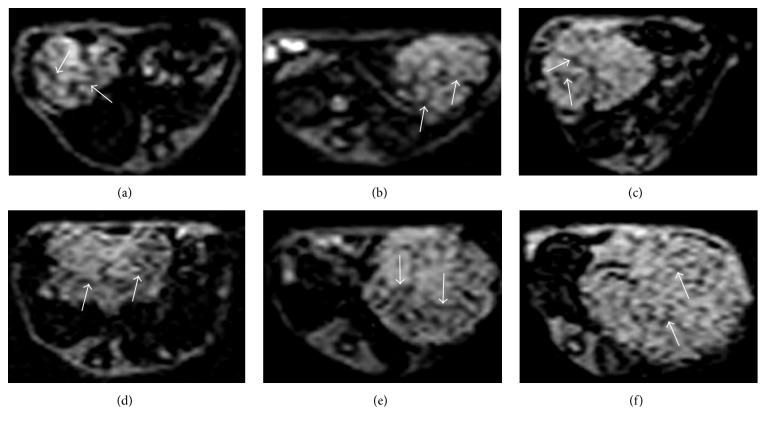
Serial measurements of intratumoral susceptibility signal intensity (ITSS) (white arrows) in the treated and control tumors on USPIO-enhanced SWI. In the treated tumors, ITSS is scored as grade 1 at 7 days (a), grade 2 at 14 days (b), and grade 2 at 21 days (c). In the control tumors, ITSS is scored as grade 3 at 7 days (d), grade 3 at 14 days (e), and grade 4 at 21 days (f). Smaller size is noted in the treated tumors than the control at each time point.

The median (P25, P75) values of ITSS scoring and mean values of *Q* for both treated and control groups at each time point were summarized in Table 1. Both ITSS (7 days,* Z *= −2.739,* P* = 0.006; 14 days,* Z *= −2.471,* P* = 0.013; 21 days,* Z *= −2.520,* P *= 0.012) and* Q* (7 days,* Z *= −2.611,* P* = 0.009; 14 days,* Z *= −2.611,* P* = 0.009; 21 days,* Z *= −2.449,* P* = 0.014) values were significantly lower in the treated group than in the control group at each time point (Figure 2).

### 3.2. Serial Measurements of ITSS Scoring and* Q* in the Treated and Control Groups

Results for serial measurements of ITSS scoring (Figure 2) and* Q* from both the treated and control groups were summarized in Table 1. There were significant changes found in both ITTS (*χ*^2^* = *11.858,* P* = 0.008) and* Q* (*χ*^2^* = *8.882,* P* = 0.031) in the treated group. ITTS scoring in the treated group persisted at 7 days (*P* = 0.699) but increased at 14 days (*P* = 0.043) and increased significantly at 21 days (*P* = 0.009) compared with the values at baseline. A significant increase in ITTS scoring was also found at 21 days versus 7 days (*P* = 0.008).* Q *was found significantly declined at 7 days (*P* = 0.028). Then,* Q* significantly recovered at 14 days (*P* = 0.028) and 21 days (*P* = 0.014) compared with 7 days.

There were significant changes found in ITTS scoring in the control group (*χ*^2^* = *15.178,* P* = 0.002). ITTS scoring significantly increased at 7, 14, and 21 days compared with the values at baseline (*P* = 0.007, 0.006, and 0.005). It was found significantly higher at 14 days and 21 days versus 7 days (*P* = 0.042 and 0.015).* Q* in the control group increased too from 7 days to 21 days, and significant difference was found among all different time points (*χ*^2^ = 9.251,* P* = 0.026). Compared with baseline,* Q *was significantly higher at 14 and 21 days (*P* = 0.016 and 0.016).

### 3.3. Effect of Sorafenib on Tumor Volume and MVD

The tumor volume at baseline was 0.440 ± 0.089 cm^3^.  Significantly smaller tumor volume was found in the treated group than the control group at each time point (7 days, 0.458 ± 0.141 cm^3^ versus 1.658 ± 0.588 cm^3^,* P *= 0.009; 14 days, 0.695 ± 0.219 cm^3^ versus 2.584 ± 0.931 cm^3^,* P* = 0.009; 21 days, 1.320 ± 0.112 cm^3^ versus 4.820 ± 0.856 cm^3^,* P* = 0.009). The tumor volume in the treated group was stable at 7 days (*P* = 0.916) and 14 days (*P* = 0.076) but increased significantly at 21 days (*P* = 0.009) as compared to the baseline.

The MVD at baseline was 25.345 ± 2.473. Significantly lower MVD was found in the treated group than the control group at each time point (7 days, 18.044 ± 3.667 versus 32.622 ± 4.876,* P *= 0.009; 14 days, 21.289 ± 3.117 versus 37.911 ± 3.001,* P* = 0.009; 21 days, 23.034 ± 3.909 versus 39.667 ± 4.451,* P* = 0.009). The MVD in the treated group was shown significantly decreased at 7 days as compared to the baseline (*P* = 0.009) but slightly recovered at the following days.

### 3.4. Correlation of ITSS Scoring and *Q* with Histologic MVD and Tumor Volume and of ITSS Scoring with *Q*

**Figure 3:**
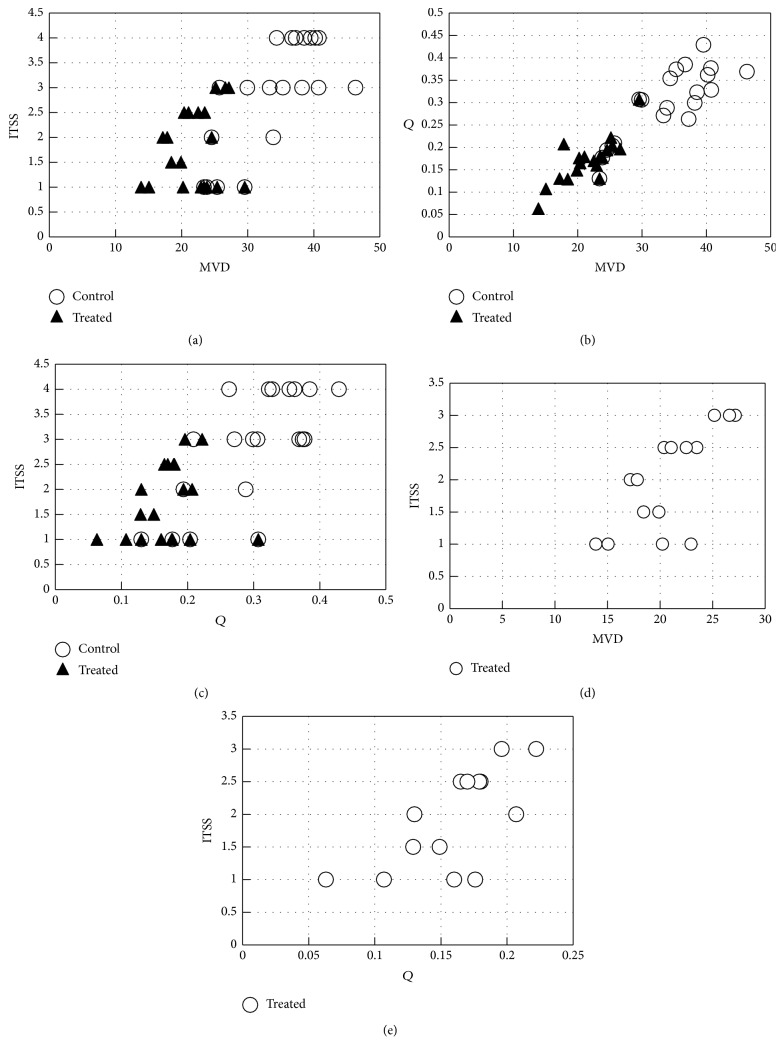
Scatterplots show the correlation between intratumoral susceptibility signal intensity (ITSS) scoring,* Q,* and microvessel density (MVD). (a) Poor correlation is shown between ITSS scoring and MVD in the treated group when all data are pooled (*P =* 0.261). In the control group, ITSS scoring shows significantly positive correlation with MVD (*r *= 0.722,* P *< 0.001). (b)* Q* demonstrates good positive correlation with MVD in both treated (*r *= 0.753,* P <* 0.001) and control group (*r *= 0.780,* P *< 0.001). (c) For the treated group, there is no correlation between ITSS scoring and* Q *when all data are included (*P =* 0.151). Good positive correlation is observed between ITSS scoring and* Q* in the control group (*r *= 0.637,* P *= 0.003). ((d), (e)) Good positive correlation exists between ITSS scoring and MVD (*r *= 0.742,* P *= 0.002) and between ITSS and* Q* (*r *= 0.713,* P *= 0.004) when data from baseline are excluded in the treated group.

In the treated group, there was poor correlation found between ITSS scoring and MVD when data from all subgroups were pooled (*P =* 0.261). However, a good positive correlation existed if data from baseline were excluded (*r *= 0.742,* P *= 0.002). *Q* demonstrated good positive correlation with MVD (*r *= 0.753,* P <* 0.001) in the treated group. In the control group, both ITSS scoring and* Q *showed significantly positive correlation with MVD (ITSS scoring:* r *= 0.722,* P *< 0.001; *Q*:* r *= 0.780,* P *< 0.001) (Figure 3).

In the treated group, ITSS scoring and *Q* demonstrated good (*r *= 0.734,* P *< 0.001) and moderate (*r *= 0.521,* P *= 0.022) correlation with tumor volume, respectively. In the control group, good correlation was observed between ITSS scoring and tumor volume (*r *= 0.807,* P *< 0.001) and between *Q* and tumor volume (*r *= 0.636,* P *= 0.003).

For the treated group, there was no correlation found between ITSS scoring and* Q* when all data from baseline to 21 days were included (*P =* 0.151), but a good positive correlation was demonstrated after exclusion of the data from baseline (*r *= 0.713,* P *= 0.004). Good positive correlation was observed between ITSS scoring and* Q* in the control group (*r *= 0.637,* P *= 0.003) (Figure 3).

### 3.5. Distribution of Iron Particles in the Tumor after Treatment

**Figure 4:**
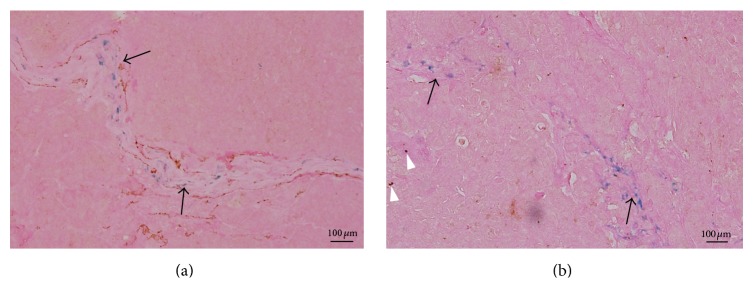
Two pathological images of treated hepatocellular carcinoma at 7 days. (a) Anti-CD31 immunohistochemistry with USPIO particles counterstaining shows blue iron particles within tumor vessels (black arrows) (magnification ×200). (b) Anti-CD68 immunohistochemistry with USPIO particles counterstaining shows several brown stained macrophages (white arrow heads) and blue iron particles within the structures (black arrows) which are consistent with positively stained vessels on anti-CD31 staining (magnification ×200).

Histologically, large amounts of iron particles were found within tumor vessels. Several macrophages were seen sparsely distributed in the tumor and few of them engulfed iron particles (Figure 4).

### 3.6. Inter- and Intraobserver Agreement on ITSS Scoring and *Q*

High inter- and intraobserver agreements for ITSS scoring and* Q* measurements in the treated group and baseline were observed and listed in Table 2. ICCs for intraobserver agreement on histologic MVD of all 35 mice were 0.937 (*P* < 0.001).

## 4. Discussion

It is well accepted that tumor vessels are heterogeneous and formed by two distinct processes, that is, early angiogenesis and late formed vessels, which mostly correspond to micro- and macrotumor vessels, respectively [10, 31]. Each of them is an indispensable part of a functional vascular network in the tumor. To monitor these tumor vascularization processes is critical especially following antiangiogenic therapy. Compared with normal vessels, the vascular wall of intratumoral vessels is morphologically and biologically immature, resulting in their much higher permeability than normal vessels [2]. Hence, MR imaging methods with macromolecular MR contrast agents, such as USPIO, which can stay in the blood with prolonged circulation time, as demonstrated in our histologic examination, are proposed to evaluate the micro- and/or macro-intratumoral vessels [11, 14, 28, 29, 32].

Although Sorafenib is the only drug which has indication for advanced HCC, it does not improve the prognosis in all advanced HCC patients [5]. It is critical to identify some imaging biomarkers that may predict the efficacy of Sorafenib treatment at an early stage, because this helps in selecting responsive patients shortly after the start of treatment. This may also enable a better understanding of the complex changes of HCC neoangiogenesis during Sorafenib treatment, which are incompletely understood. By using combined USPIO-enhanced SWI and* Q* in this study, we demonstrated that ITSS scoring and* Q* quantification could be used with high intra- and interobserver agreement to reflect sequential effects of Sorafenib on both macro- and microtumor vasculature of HCC, and both ITSS and* Q *were correlated well with tumor volume and histologic MVD. The antiangiogenic effects of Sorafenib were further confirmed in our xenograft models by smaller tumor volume and lower MVD in the treated than the control mice.

ITSS scoring which represents macrovessels in the tumor has been reported as a potential imaging surrogate for neovascularity in neoplasms [11, 25, 26, 33]. Serial observation of changes in ITSS has been used to monitor tumor vessel responses to antiangiogenic therapy in some clinical studies [34, 35]. In our study, although ITSS was shown to persist at 7 days after treatment and increased at 14 and 21 days, it was significantly lower in the treated than the corresponding control subgroups at each time point. Similar ITSS changes have been reported in patients with posttransplant lymphoproliferative disorder in central nervous system and in patients with malignant glioma over the courses of antiangiogenic treatments [34, 35].

*Q* has been validated as an MR estimate of histologic MVD in animal tumor models and is independent of the concentration of contrast agent [13]. However, there have been few reports so far using* Q* to track tumor microvessels during antiangiogenic treatments [14, 28]. Our present study demonstrated that* Q* correlated well with MVD in HCC xenografts in both the control and treated groups. Like ITSS,* Q* showed significant reduction in the treated group compared to the control at each time point. Repeated posttreatment measurements revealed a significant decrease of* Q* at 7 days followed by an increase at 14 and 21 days, which were in agreement with the trend of change in histologic MVD. The later increase of* Q* was speculated to be triggered by hypoxia in the tumor microenvironment resulting from initial suppression of tumor microvasculature after treatment [36]. However, the change in* Q* and ITSS at 7 and 14 days was nonsynchronous because the antiangiogenic effect of Sorafenib takes place on microvascular level which could not be initially reflected by ITSS. Only when these ‘‘early” microvessels evolved over a period of time into ‘‘late” macrovessels, could they be detected on SWI. Our quantitative results from* Q* and ITSS could provide complementary information to previous reports, in which vascular normalization after antiangiogenic therapy was considered as a possible cause resulting in the increase of vascular volume fraction in the tumor measured with MR perfusion methods [37].

In the control mice, there was positive correlation between ITSS and MVD. Furthermore, we found after initiation of Sorafenib treatment that ITSS was correlated well with MVD, though its posttreatment response might lag behind* Q *and MVD. These findings imply that ITSS could serve as a biomarker of tumor vascularity both in HCC's natural course of development and after antiangiogenic therapy.

Our study had several limitations. First, the immunohistochemical section of the tumor may not completely match the slices for SWI and* Q* evaluation. MVD counted at this section may not represent the whole tumor microvessels because tumor vasculature is heterogeneous. Second, 1 mouse at 21 days in treated group was excluded from the study because the tumor signal did not decrease after USPIO injection resulting in the negative Δ*R*_2_ values. The underlying causes remained unknown. Sampath et al. assumed that negative Δ*R*_2_ or ΔR_2_^*∗*^ values resulted from poor perfusion [28]. However, ITSS of the tumor in this mouse could still be identified. And the exclusion would not affect the correlation analysis in this group. Third, a direct comparison between ITSS and histologic macrovessels of removed HCC was not done due to technical difficulties. Although ex vivo micro-CT angiography of the tumor might be used to validate ITSS, there are still multiple issues hampering its accuracy as a reference standard [14, 28]. Fourth, because of the “blooming effect” resulting from USPIO [38], ITSS could only be semiquantitatively analyzed. It does not reflect the true size of the tumor macrovessels. Fifth, tumor microhemorrhage and USPIO swallowed by macrophages in the tumor may interfere with ITSS and* Q* evaluation, although macroscopic bleeding was ruled out by reviewing the conventional MR images and most of the USPIO particles were shown to stay intravascularly on histologic sections. Lastly, cautions should be exercised about drawing inferences from an experimental HCC model to human HCC since the effects of Sorafenib could be different and the incidence of hemorrhage identified within xenografts imaged for this study was low.

Clinical translation of USPIO-enhanced SWI and mean vessel density imaging* Q* may be expected in the future since the safety and effectiveness of off-label use of ferumoxytol, which is a USPIO product approved by the FDA for treatment of iron deficiency anemia, as an alternative MR contrast agent, were confirmed in some recent studies [39, 40]. Although further clinical investigation is warranted, currently combined USPIO-enhanced SWI and* Q* measurement may be useful as a novel preclinical therapeutic testing for newly developed antiangiogenic treatments in different tumor models.

## 5. Conclusion

Our results demonstrate that ITSS scoring and* Q* obtained from steady-state USPIO-enhanced MRI can quantitatively evaluate both macro- and microvascular responses of HCC to Sorafenib treatment. They may provide valuable information which is complementary to widely reported functional properties of tumor angiogenesis assessed by perfusion-weighted technologies.

## Figures and Tables

**Table 1:** Sequential measurements of ITSS scoring and* Q* in the treated and control groups and *P* values for comparisons between different time points.

Parameter	Treated group				Control group			
	Measurement	*P* values^a^			Measurement	*P* values^a^		
		7 days	14 days	21 days		7 days	14 days	21 days
ITSS scoring [median (P25, P75)]								
Baseline (*n* = 5)	1 to 2 [1 (1,1.5)]	0.699	***0.043***	***0.009***	1 to 2 [1 (1,1.5)]	***0.007***	***0.006***	***0.005***
7 days (*n* = 5)	1 to 1.5 [1 (1,1.5)]		0.051	***0.008***	2 to 3 [3 (2.5,3)]		***0.042***	***0.015***
14 days (*n* = 5)	1 to 3 [2.5 (1.5,2.75)]			0.381	3 to 4 [4 (3,4)]			0.513
21 days (*n* = 5)	2 to 3 [2.5 (2.25,3)]				3 to 4 [4 (3.5,4)]			

*Q* (means ± SD)								
Baseline (*n* = 5)	0.130 to 0.307 (0.203 ± 0.065)	***0.028***	0.347	1.000	0.130 to 0.307 (0.203 ± 0.065)	0.076	***0.016***	***0.016***
7 days (*n* = 5)	0.063 to 0.160 (0.122 ± 0.038)		***0.028***	***0.014***	0.209 to 0.374 (0.295 ± 0.059)		0.175	0.251
14 days (*n* = 5)	0.130 to 0.222 (0.175 ± 0.033)			0.462	0.271 to 0.385 (0.344 ± 0.047)			0.917
21 days^b^	0.170 to 0.207 (0.188 ± 0.017)				0.263 to 0.429 (0.349 ± 0.060)			

^a^Data were tested with Kruskal Wallis test followed by Mann–Whitney *U* test in case of statistical significance. ^b^There are 4 mice in the treated and 5 mice in the control group at 21 days for *Q* evaluation; ITSS, intratumoral susceptibility signal; SD, standard deviation.

**Table 2:** Intra- and interobserver agreement on ITSS scoring (*n* = 15) and *Q* (*n* = 14) in the treated group with baseline (*n* = 5).

Parameter	ITSS scoring		*Q*	
	Kappa	*P*	ICCs	*P*
Observer 1	0.640	0.000	0.938	0.000
Observer 2	0.791	0.000	0.954	0.000
Interobserver	0.732	0.000	0.906	0.000

ITSS, intratumoral susceptibility signal; ICCs, intraclass correlation coefficients.
